# The Historical Distribution of Main Malaria Foci in Spain as Related to Water Bodies

**DOI:** 10.3390/ijerph110807896

**Published:** 2014-08-06

**Authors:** Arturo Sousa, Leoncio García-Barrón, Mark Vetter, Julia Morales

**Affiliations:** 1 Department of Plant Biology and Ecology, University of Seville, E-41012 Seville, Spain; E-Mail: jmorales@us.es; 2 Department of Applied Physics II, University of Seville, E-41012 Seville, Spain; E-Mail: leoncio@us.es; 3 Faculty of Information Management and Media, Karlsruhe University of Applied Sciences, Moltkestr. 30, D-76133 Karlsruhe, Germany; E-Mail: mark.vetter@hs-karlsruhe.de

**Keywords:** malaria, spatial analysis, Spain, water bodies, climate change, wetlands, Geographic Information Systems (GIS)

## Abstract

The possible connectivity between the spatial distribution of water bodies suitable for vectors of malaria and endemic malaria foci in Southern Europe is still not well known. Spain was one of the last countries in Western Europe to be declared free of malaria by the World Health Organization (WHO) in 1964. This study combines, by means of a spatial-temporal analysis, the historical data of patients and deceased with the distribution of water bodies where the disease-transmitting mosquitos proliferate. Therefore, data from historical archives with a Geographic Information System (GIS), using the Inverse Distance Weighted (IDW) interpolation method, was analyzed with the aim of identifying regional differences in the distribution of malaria in Spain. The reasons, why the risk of transmission is concentrated in specific regions, are related to worse socioeconomic conditions (Extremadura), the presence of another vector (*Anopheles labranchiae*) besides *A. atroparvus* (Levante) or large areas of water bodies in conditions to reproduce theses vectors (La Mancha and Western Andalusia). In the particular case of Western Andalusia, in 1913, the relatively high percentage of 4.73% of the surface, equal to 202362 ha, corresponds to wetlands and other unhealthy water bodies. These wetlands have been reduced as a result of desiccation policies and climate change such as the Little Ice Age and Global Climate Change. The comprehension of the main factors of these wetland changes in the past can help us interpret accurately the future risk of malaria re-emergence in temperate latitudes, since it reveals the crucial role of unhealthy water bodies on the distribution, endemicity and eradication of malaria in southern Europe.

## 1. Introduction

Spain was one of the last countries of Western Europe in which malaria was declared officially eradicated, in 1964, by the World Health Organization (WHO) [1]. Other European countries managed to eradicate autochthonous malaria not long after the Second World War [2,3], such as Germany in 1950, Holland in 1961, Italy in 1970, and Portugal and Greece in 1973 [4]. More recently, the WHO has reported cases of malaria in Georgia, Azerbaijan, Kyrgyzstan, Tajikistan, Uzbekistan and Turkey [5], and there have been sporadic locally acquired cases in Mediterranean European countries like France, Italy, Greece [5] and Spain [6].

Several factors influences the distribution of malaria: hygienic-sanitary conditions, social-economic situations, land use changes, demographic mobility, malaria prophylaxis programs, *etc.* These factors may influence the development of mosquito species that act as malaria vector. However, they can also influence the development of the parasites that cause the disease. Historically, wetlands have been linked to the presence of malaria, especially in endemic areas. In Spanish archives, an accumulation of water bodies, where the *Anopheles* species can grow up is translated to “unhealthy water bodies”, in Spanish “Colecciones líquidas insalubres”. In Spain, a legal regulation was created with the aim of desiccating these wetlands, like the Law of Cambó, on the 24 July 1918 [7], or the Waters Law of 1866 and 1879. In fact, the original word for malaria in Spanish (“Paludism”) is from the Latin word “Palus” (swamp, pool) [8]. At present, the involvement of malaria with global climate change is under discussion and it is considered to be among the infectious diseases with risk of re-emergence in temperate areas where it was eradicated [9,10,11]. In temperate regions, malaria is considered a potentially re-emerging disease due to changes in environmental conditions, temperature, land use changes, and to the increase of tourist flows from outside endemic areas [12]. We believe that knowing the factors that conducted the eradication of malaria in Western Europe and particularly in Spain may help in better understanding the factors that influence the risk of re-emergence in temperate countries due to global climate change. A better understanding of these processes regulating the distribution of unhealthy water bodies helps us to calibrate the driving forces for climate scenarios in the future. Furthermore, Spain, due to its proximity to North Africa, has an additional interest. Hence, predictions (for 2050 in Spain) do not reflect a transmission scenario in terms of increasing disease risk, otherwise it is predicted for the Moroccan coast [1]. In this sense, we cannot rule out the possibility that African vectors susceptible to tropical *Plasmodium* strains could invade the southern part of the Iberian Peninsula [1].

Some authors state that there are regions in Spain with greater endemic prevalence of autochthonous malaria than others [13,14]. Periodic fluctuations would overlap with this basic pattern until epidemic characteristics are reached in the whole country, as it happened in the 18th century [15,16] or after the Spanish Civil War in the 20th century [13,17]. In order to test this idea, we are going to combine the techniques of the Geographical Information System (GIS) with the data from historical archives. If it is possible to establish regional differences in the distribution of autochthonous malaria in Spain we will determine whether the presence of flooded areas plays an important role in these differences. Therefore, the main objectives are to (1) analyze the historical evolution of autochthonous malaria in Spain in the 20th century; (2) investigate the regional and/or provincial distribution of malaria in Spain to establish whether all areas show homogenous behavior; (3) analyze the spatial distribution of unhealthy water bodies and determine if there is any linkage with the historical cores of greater endemic malaria; and (4) analyze the effects of the trends of climate change on the area occupied by unhealthy water bodies.

## 2. Material and Methods

In order to reach these objectives we are going to analyze in a first step the secular tendencies for the distribution of autochthonous malaria cases during the 20th century in Spain. As a second step, we focused on the spatial distribution of foci with higher endemicity of the disease from available provincial data from the historical archives. In the third part, we mapped the regional data of water bodies regarding its spatial distribution in comparison to areas with focal points of malaria transmission. Finally, we studied the climatic tendencies in the areas of higher malaria transmission points and their relation with the area occupied by unhealthy water bodies. An overview of the applied methodology and data can be seen in Table 1.

**Table 1:** Overview of the applied methodology and data of the study.

Objectives	Data	Spatial extension	Methods
Historical evolution of autochthonous malaria	Database of the Spanish Statistical Institute	Spain	Mann-Kendall-Test
Spatial distribution of malaria in Spain (death and diseased persons)	Database of the Spanish Statistical Institute	Spain (province)	Inverse Distance Weighting (IDW interpolation)
Spatial distribution of unhealthy water bodies	Database of the Spanish Statistical Institute	Spain (regional)	Geographic distribution of the percentage and total surface occupied by unhealthy water bodies
Trends of climate change	Daily maximum and minimum temperatures, average minimum temperatures and spring rainfall	Southwestern Spain	Inter-annual distribution of the minimum and maximum average temperatures, inter-annual variations of the minimum average temperatures, and inter-annual variation of spring rainfall in the southwest of Spain in the 20th century

**Figure A1:**
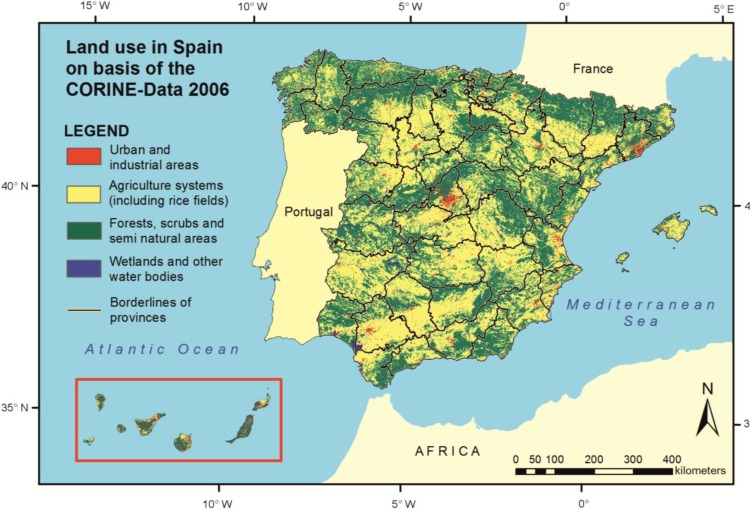
Map of land use in Spain on basis of the CORINE-Data 2006.

Even if in ancient times the land use unit areas in Spain were probably different, we added a map to the Supplementary Material (see Figure A1) in order to get an idea about the distribution of water bodies and wetlands, as well as major recent land use activities in Spain.

### 2.1. Trends of Autochthonous Malaria in Spain throughout the 20th Century

Since it is a notable infectious disease, there is a vast data bank in the historical archives of the Spanish Statistical Institute. Thereby, in order to quantify the evolution of malaria in Spain throughout the 20th century, we performed a thorough review of the database of the Spanish Statistical Institute (SSIbase) [18]. These archives correspond to annual reports or yearbooks, which began in 1858 in pursuance of the organic regulation of the general Statistics Commission of Spain. The first complete epidemiological data for all of Spain (considering the current international borders), appears in 1900. The word used for malaria varies among the yearbooks of the historical archives of the SSIbase. In the yearbooks between 1861 and 1864 there are references to the symptoms rather than to the etiology (“Simple intermittent fevers” and “Malignant intermittent fevers”). From the early 20th century until 1930 these are known as “intermittent fever” and “malarial cachexia”, and from that date they appear under the heading of “Paludism” (Malaria). With these data, we obtained the evolution—in absolute numbers—of annual deaths by autochthonous malaria in Spain throughout the 20th century (1901–1959), besides the total annual number of deaths regardless of their cause.

From the total number of deaths by malaria we have analyzed the seasonal trends for the period of 1901–1959. With the aim of determining the significance of the seasonal trends, a Mann-Kendall-Test was applied to estimate the *Q* Sen’s value in order to detect the gradient of monotonic trends [19] using MS Excel spreadsheet [20]. In the seasonal analysis, the trends due to the general improvement of hygiene-health and socioeconomic conditions were separated from the trends that correspond to seasonal fluctuations. In this calculation, we excluded two brief periods that represent an anomalous, odd behavior (those corresponding to years of war and/or port war periods) during which the general trend is disrupted. The variable obtained (*P_m_*) indicates the number of deaths by malaria each year per every one thousand deaths in that same year. We can consider that this variable is composed of two components (Equation (1)):

*P_m_ = p_m_ +* δ (1)

A quadratic component *p_m_* which justifies most of the variance of the period between 1901 and 1959 (*p_m_ = at*^2^ *+ bt + c*, where *t* represents the year) (Equation (2)) and a residual component (δ).

The residual component (δ) is obtained as the difference between the actual values of each year (*P_m_*), calculated from the data of mortality registered each year, and the corresponding theoretical values (*p_m_*)

*p_m_ = 0.0045t*^2^*– 0.3687t + 7.4661* (2)

obtained through the polynomial fit of the seasonal sets. Depending on whether the actual values *P_m_* are higher or lower than the theoretical values *p_m_*, the annual residues *δ* will be positive or negative, respectively. Component *p_m_* is associated to permanent conditions like the progressive improvement of the hygiene-health conditions throughout the years. On the other hand, the residual component, without a significant seasonal trend, is associated to seasonal fluctuations like climatic variations, agrifood variations, *etc*.

### 2.2. Spatial Distribution of Autochthonous Malaria in Spain in the 20th Century

In some particular newspapers of the 20th century, the annual reports on the SSIbase show more detailed information about the disease. There are disincorporated data by province about the number of deaths for the period between 1916 and 1930, and the number of diseased people between 1949 and 1954 and 1961. In the latter period, there is also the monthly distribution of the number of diseased people in all of Spain.

**Figure A2:**
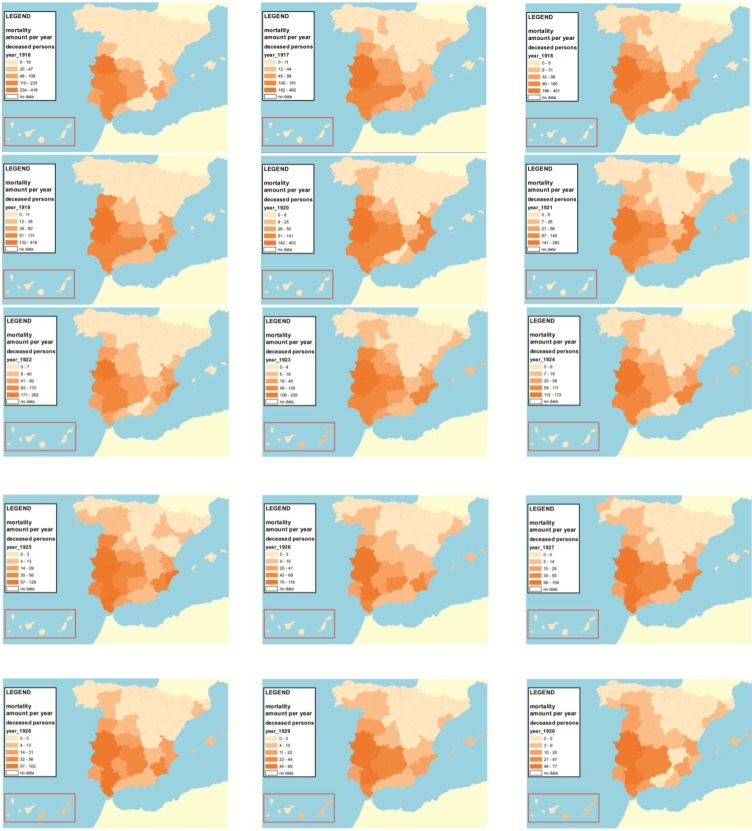
Maps of the distribution of the number of deaths people by malaria in each of the years. (1916–1930) by province.

**Figure A3:**
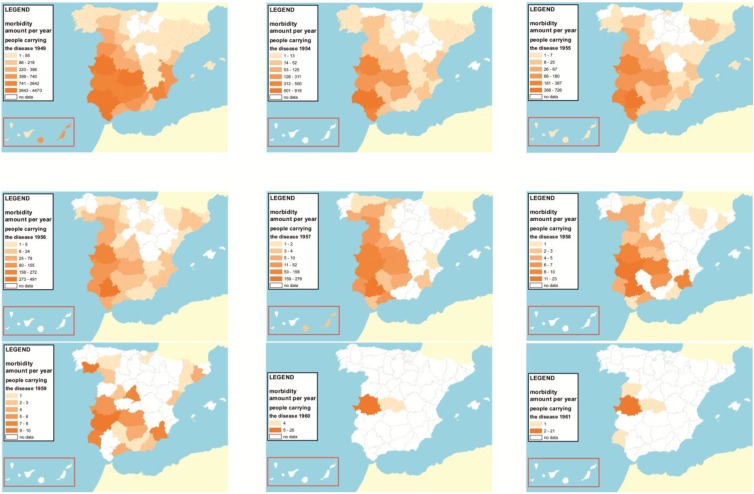
Maps of the distribution of the number of diseased people by malaria in each of the years (1949 and 1954-1961) by province.

These data were used to obtain a mapping of the distribution of the number of deaths and diseased people by malaria in each of the years of which there are disincorporated data by province (see Figure A2 and Figure A3). In order to spatially visualize this information and obtain a mapping for the whole country we used GIS-techniques that have emerged as the core of the spatial technology which integrates wide range of dataset available from different sources [21]. In the last few years, spatial approaches has been used in ecoepidemiological studies of malaria [22,23], in anopheles vector mapping [24], and in risk, control and prevention of this disease [25]. It can also be used to obtain historical maps of malaria in different regions across the globe [26]. In order to realize the aims of our study, we used a GIS for the purposes to summarize pointwise data, to bring this information into a spatial context by geostatistical functions and finally to design the maps. The main aim in the context was to visualize the spatial patterns in order to provide help to find a hypothesis. Using this above mentioned GIS-based methods we obtained maps with different surface characteristics separated by isolines of the average annual number of deaths (1916–1930) and diseased (1949 and 1954–1961) of autochthonous malaria in Spain. In order to compile the maps the centroids of the Spanish provinces were calculated on the basis of the province geometric middle point of the area within the administrative borders by the ESRI ArcGIS (ArcGIS for Desktop Advanced Version 10.1 with extension Spatial Analyst). To combine these geodata of the provinces, the shape files were joined with the data table of the average number of all years of the study period of the cases of deaths (1916–1930) and diseased people (1949–1961). To visualise the spatial distribution, the geostatistical Inverse Distance Weighting (IDW interpolation) was applied. Inverse Distance Weighting (IDW) is statistically a deterministic method for multivariate interpolation with a previously known scattered pattern of points. The assigned values to unknown points are calculated with a weighted average of the values which corresponds to the already known points. The denomination given to this type of method was inspired by the weighted average applied since it resorts to the inverse of the distance to each known point (“amount of proximity”) when assigning weights. The set of parameters was chosen by several attempts to receive finally the best cartographic visualization. This technique was used to study environmental relations with other infectious diseases [27], since it can produce maps by interpolating dispersion points (more influenced by the closer points and less by those more distant). As parameters for the IDW interpolation method we used for the generation of both maps in Figure 2 the power value of 8, and the number of used neighbor values was 12. Even if in other studies the power value of 2 is used, we choose the above mentioned parameters as a result of several attempts in order to find the best spatial representation of the phenomena according our study.

It should be remarked, that the map in Figure 2a,b does not reflect the real spatial distribution of the malaria cases, it just shows a spatial summarized distribution approximation of the phenomena for a longer period (several years). This was carried out to visualize, that the extreme spatial differences happens not only in particular years. We are aware that this aggregation is influenced by the modifiable areal unit problem (MAUP). This needs to be considered by the interpretation of the maps in the Figure 2a,b. In order to visualize the real provincial based distribution of each year in the study area, we added as Supplementary Material further maps (see Figure A2 and Figure A3).

### 2.3. Unhealthy Liquid Collections and Malaria in Spain in the Early 20th Century

Besides the data corresponding to the number of diseased people and deaths by autochthonous malaria, we have reviewed in the SSIbase other data that may be related to the disease; specifically, the SSIbase 1915 and SSIbase 1917 yearbooks, which provide information at a regional scale about the disease and other economic and environmental variables. These data are from the preview-abstract of statistical data of paludism in 1913 and 1916 in Spain, published in the yearbooks of 1915 and 1917, of the Field Health inspection (Directorate General of Agriculture). These two yearbooks include information of the area of “foci” (above mentioned unhealthy water bodies) of paludism in hectares, clarifying that these are flooded lands that require sanitation in order to prevent them from becoming infectious foci. These lands exclude—as quoted by the inventory preview mentioned—“those foci constituted by ricefields, hemp rafts, riverbanks, channeled streams, gutters and railroad trenches”. Therefore, they only refer to unhealthy water bodies as they are potentially suitable for the reproduction and growth of the vector that transmits malaria.

These two inventories also include data of the number of malarial municipalities with respect to the total number of municipalities in each region, regarding as such those in which paludism is permanent. Furthermore, they gather data of morbidity, mortality, up-to-date value of malarial lands, approximate cost of sanitation works, days of work lost by paludism, annual consumption of quinine, *etc.* From these data we have analyzed the correlation among the different variables and whether the relation between them is or not similar in all the Spanish regions. Since we have two complete years and 13 differentiated regions, the initial sample size for the comparison is *n* = 26, although the data of the Canary Islands are not complete.

Moreover, the monthly distribution of the number of diseased people was calculated for the years in which there is information available (1949 and 1954–1961) in percentage. Furthermore, the polynomial function was calculated for all the years in order to determine whether the intra-annual distribution of diseased people has a constant pattern associated to the seasonal climate variables. These data were compared with the intra-annual distribution of the daily maximum and minimum temperatures in the observatory of Seville-Airport (Southwestern Spain) throughout the period between 1951 and 2001 [28].

Finally, based on previous studies, we elaborated climate sets to analyze the increase of the average minimum temperatures and the variation of the spring rainfall throughout the 20th century. In order to do this, we calculated the mean value of the spring rainfall recorded in the observatories of Southwestern Spain with long pluviometric sets: Córdoba, Seville, San Fernando (Cádiz) and Riotinto (Huelva), since the southwest of Spain is a region with many wetlands [29] and it is also historically considered as a severely endemic area [3]. The weather station of Seville was used for the seasonal analysis of the average minimum temperatures. Our aim is to establish some direct or indirect linkage between the climate variables and some of the variables related to malaria, not only to see their impact in the spreading of the disease, but also to determine whether the climate trends in the 21st century may influence or favor the re-emergence risk of introduced malaria.

For the province of Huelva, one of the most severe endemic areas of malaria in Spain, with a vast surface covered by wetlands, different texts from the mid-20th century have been compiled, which were elaborated by the people in charge of the desiccation of coastal lagoons. These are included in Table A1.

## 3. Results and Discussion

First of all, we present the results of the historical evolution of malaria in all of Spain throughout the 20th century. Then, we analyze the spatial distribution of the disease at a provincial and regional level, and its relation with the unhealthy liquid collections. Finally, we analyze the potential relations between these unhealthy liquid collections and the recent climate trends.

### 3.1. Autochthonous Malaria trends in Spain throughout the 20th Century

**Figure 1:**
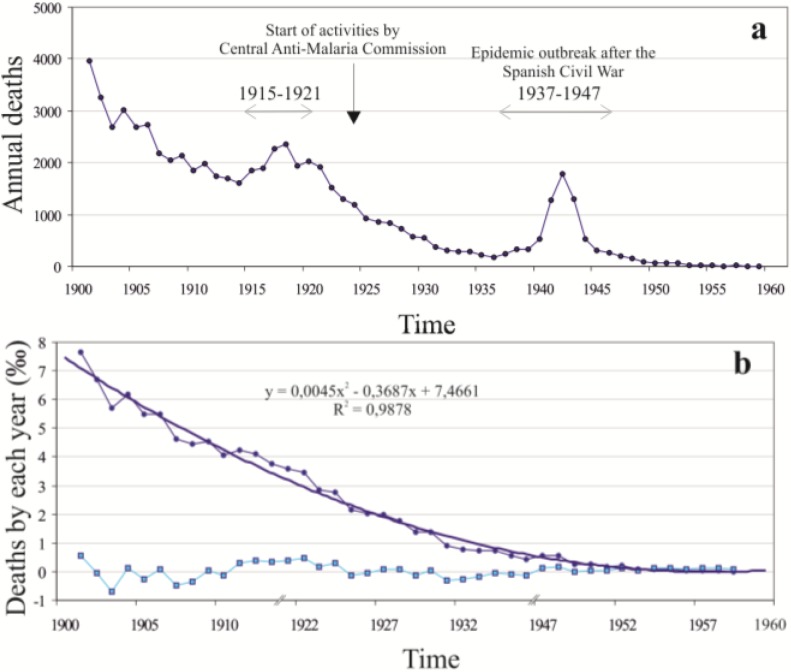
(**a**) Evolution of mortality by autochthonous malaria in Spain. (**b**) Proportion of deaths by malaria in relation to the total mortality between 1900 and 1959 (the years 1915–1921 and 1937–1947 are not represented).

After applying the Mann-Kendall-Test for the trends of the annual number of cases for the malaria mortality in Spain (1900–1959) we have found a significant trend (*n* = 59, *p* < 0.001, slope (*Q*) = −50.8). This sharply downward trend shows two saw teeth (Figure 1a) in which the number of deaths increases notably (1915–1921 and 1937–1947). The improvement in hygienic-social conditions and the creation in 1924 of the Central Anti-malaria Commission contribute to maintain a decrease, only interrupted by the Spanish Civil War (1936–1939) and the post-war period. It was statistically proved by the Mann-Kendall-Test that the trends of decrease (negative slope (*Q*) value) of deaths (1901–1959) by malaria in Spain considering the annual average values are significant. In order to determine whether the general improvement of hygiene-health and socioeconomic conditions in the 20th century accounts for this trend, we analyzed the mortality caused by malaria in relation with the total mortality in Spain. To do this, discarding the two saw teeth of 1915–1921 and 1937–1947, we calculated the proportion of deaths by malaria with respect to the total of deaths (as per thousand). The result obtained may be represented as two summands (Figure 1b): first, a quadratic component that accounts for 98% of the variance of the period between 1901 and 1959 (*p_m_= 0.0045t^2^ − 0.3687t + 7.4661*), which may be associated to constant or permanent causes, presumably due to the progressive improvement in hygiene-health and social conditions; and second, a residual component associated to more fluctuating, variable causes, like climate trends, agrifood production, *etc*.

After the Spanish Civil War there was an important upturn of paludism in Spain, being 1941, 1942 and 1943 the years with greater mortality, as during these the number of deaths by paludism is three times higher. During 1941, malaria was the number 11 cause of mortality in all of Spain [14]. In the case of Delta del Ebro, although this upturn coincides with an increase of surface occupied by ricefields, this does not seem to have been the cause for this new epidemic outbreak [30]. The data obtained from SSIbase show that, from the 1950s in Spain, many diseases decreased in their morbidity (for example, typhoid fevers, pulmonary tuberculosis or measles), although the most marked decrease occurred in malarial fevers. During the mid-1950s, the Spanish Southwest (more specifically Seville, Huelva and Cádiz, in that order) accumulated the highest number of cases of paludism of the whole country. This makes us consider Western Andalusia as an important focus of endemic malaria. In 1959, the last death by autochthonous malaria took place in Spain, and in 1961 the last people diseased by malaria were declared in the provinces of Cáceres, Huelva, Salamanca and Toledo [14].

Another different aspect is whether these trends are homogenous in Spain or they rather show a differentiated behavior in certain geographic areas where the number of diseased people and/or deaths is higher. Should the distribution not be homogenous, this could indicate that regional or provincial factors linked to their spatial or geographical situation are also influencing the trends.

### 3.2. Spatial Distribution of Autochthonous Malaria in Spain throughout the 20th Century

**Figure 2:**
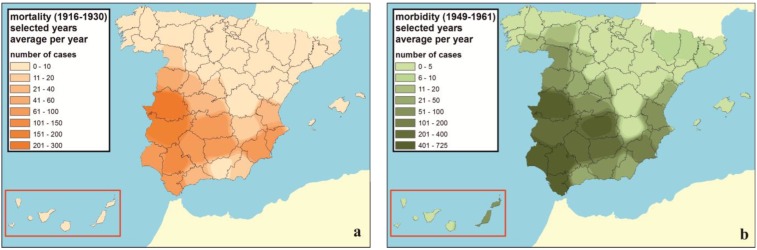
(**a**) Provincial geographical distribution of the average number of deaths by malaria throughout the period between 1915 and 1930. (**b**) Average number of diseased people in 1949 and between 1954 and 1961.

The tools for spatial-seasonal analysis provided by the techniques supported by a GIS allow expressing epidemic relations and risk areas [27], which are useful for understanding the spatial distribution of the disease. The average annual distribution of the number of deaths (1915–1930) and the number of diseased people (1949–1961) shows a behavior differentiated by zone. It should be stated again, that these maps are only a summarized visualization of the phenomena regarding several years. Detailed maps for the spatial distribution in each year could be found in the Supplementary Material S2. The highest mortality levels (Figure 2a) take place in the regions of Extremadura (especially in Cáceres), Western Andalusia and on the Mediterranean coast (specifically in Levante and Murcia). With the number of diseased people in the mid-20th, century similar results are obtained (Figure 2b), the highest being in the provinces of Cáceres, Ciudad Real, Cádiz, Seville, Huelva and Murcia.

From 1903 until 1918, paludism affected preferentially Extremadura, Murcia, Andalusia, Toledo and Ciudad Real [13,31]. This distribution was stable in a similar manner, even during the outbreak of the Spanish post-war period [13]. These results are consistent with the mapping elaborated in 1933 by Hernández Pacheco for Spain, who regards as severely endemic areas the regions of Extremadura, the Betis valley and the crops of Alicante and Murcia [3]. According to Hernández Pacheco, the Hills of Toledo and Sierra Morena had intense endemic character, and the Mediterranean coast and the Castilian plateau were regarded as slightly endemic. What causes these differences among Spanish provinces both in mortality and morbidity by malaria? In order to answer this question we analyzed jointly the data shown in the yearbooks of 1913 and 1916. Within these, besides the number of diseased people and deaths for these two years, there are complementary data of interest, a synthesis of which is shown in Table 2.

**Table 2:** Mean values of environmental and sanitary variables related to malaria in Spanish regions in 1913 and 1916.

Regions	Nº of malarial municipalities affected	Size of unhealthy water bodies (ha)	Consumption of quinine (gr)	Estimated economic loss (pesetas)
*Castilla la Nueva*	96.5	441.0	50,062.5	647,128.5
*La Mancha*	93.5	58,131.5	72,830.0	7695,457.5
*Extremadura*	332.0	2146.5	718,612.5	8451,034.3
*Castilla la Vieja*	206.5	1761.5	59,095.0	1340,226.4
*Aragón and Rioja*	10.0	321.0	3425.0	264,190.0
*Leonesa*	185.0	4843.5	47,924.0	1502,548.6
*Asturias and Galicia*	15.0	6722.0	4720.0	31716,671.3
*Navarra and Vascongadas*	21.0	59.0	1147.0	80,329.8
*Cataluña*	57.0	2944.0	30,600.0	1905,202.8
*Levante*	201.0	33,445.0	459,804.0	5097,987.5
*Eastern Andalusia*	70.5	3934.0	181,076.5	3749,531.3
*Western Andalusia*	176.0	208,527.0	817,250.0	47927,435.0
*Baleares*	9.0	3857.0	15,600.0	275,350.0
*Canarias*	--	--	--	--
TOTAL	1473	327,133	2462,146.5	110653,093.0

The results in Table 2 allow estimating that in the early 20th century in Spain there was an average annual need for 2.5 t of quinine, and that malaria involved a loss average annual of 111 million pesetas to the country from 1913 to 1916 (the equivalent in 1913 and 1916, to 875 and 347 million euros in 2014, respectively). Furthermore, a total of 1473 Spanish municipalities were affected by paludism and it was estimated that an average of 327,133 ha were occupied by unhealthy water bodies. After analyzing the data in Table 2, it is obvious that the distribution of these unhealthy water bodies was not homogenous in the whole country.

### 3.3. Unhealthy Liquid Collections and Malaria in Spain in the Early 20th Century

**Figure 3:**
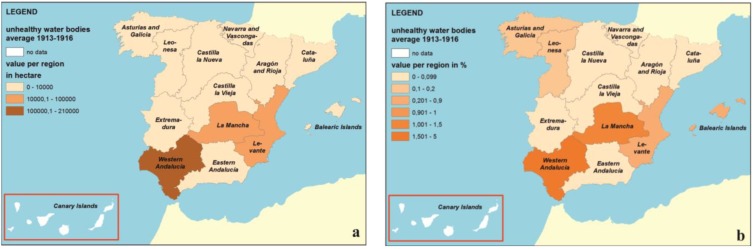
(**a**) Geographic distribution of the total surface occupied by unhealthy water bodies in each region. (**b**) Percentage of the surface occupied by unhealthy water bodies in each region.

The results in Table 2 also allow elaborating maps of unhealthy liquid collections in the different regions of Spain between 1913 and 1916. If we represent the percentage of surface occupied by unhealthy liquid collections with respect to the total surface of each region (Figure 3), we can obtain a global image of their distribution with a comparative view.

Figure 3a highlights Western Andalusia, where the greatest surface of unhealthy water bodies of Spain in 1913 is concentrated (208,527 ha de un total de 327,133 ha). Other regions with large areas are La Mancha (58,131 ha) and Levante (33,445 ha). With respect to their distribution, most of these unhealthy water bodies (87%) were situated in Western Andalusia (68%) and La Mancha (19%). The rest of the Spanish regions showed, each of them, a surface below 3% of the total occupied by the masses of unhealthy water bodies in all of Spain. In this analysis it is convenient to consider that the total surface occupied by each of the different regions is very variable. Therefore, Figure 3b shows the percentage of each region occupied by unhealthy water masses with respect to the surface of each region. Western Andalusia stands out also in this figure, with 4.6% of its surface occupied by unhealthy water bodies in the early 20th century. La Mancha reached 1.4% of its regional surface, followed by Levante (0.9%) and Baleares (0.8%); the rest of the regions did not overpass 0.2%.

These results are in line with Figure 2a,b, although Extremadura shows greater number of diseased people (71,658 in 1913), and especially deaths (833 in 1913), considering its average surface of unhealthy liquid collections (2146 ha) and the scarce percentage of its surface (0.1%). This can be compared with the number of deaths in 1913 in Western Andalusia (706), Levante (289) or La Mancha (89).

**Figure 4:**
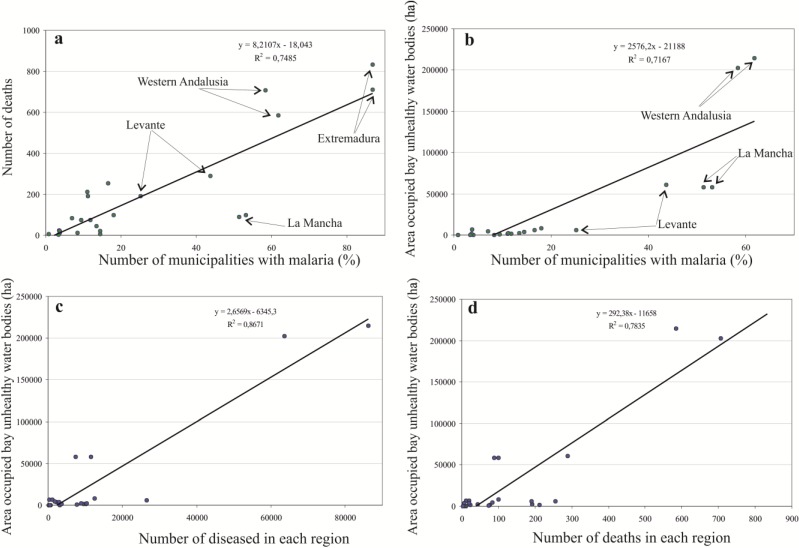
(**a**) Correlation between the number of deaths and the percentage of malarial municipalities. (**b**) Correlation between the area of unhealthy liquid collections and the percentage of malarial municipalities. (**c**) Correlation between the area of unhealthy liquid collections and the number of diseased people. (**d**) Correlation between the area of unhealthy liquid collections and the number of deaths.

In order to go into detail about the different distribution of diseased people, deaths and the distribution of unhealthy wetlands in Spain, we analyzed the correlation among the different hygiene-health and environmental conditions shown in the original tables of the yearbooks of 1913 and 1916 by calculating the coefficient of determination R-sq and representing in diagrams the dispersion of the different variables listed in Table 2 for each region (Figure 4).

As it would be expected, there is a correlation between the number of diseased people and deaths and the percentage of municipalities with malaria with respect to the total of municipalities in each region (R-sq 0.75 for the number of deaths and 0.67 for the number of diseased people). Andalusia, Levante and Extremadura show greater mortality in the early 20^th^ century than the rest of the country regarding the percentage of municipalities with malaria. A factor that may influence this, is the rural character and/or lesser socioeconomic development in some of these regions. The data of the historical yearbooks of 1955 and 1960 reveal that mid-20th century malaria showed a markedly rural distribution in Spain. In the case of Southwestern Spain, this could be related to the proximity of lagoons and other flooded areas, which would have served as reservoirs for the vector that transmits the disease [14]. In fact, of the 3791 cases of paludism in the year 1954 only 48 (1.26%) corresponded to the capital cities of the Spanish provinces. In 1955, 2514 cases were recorded, of which only 20 (0.79%) corresponded to capitals of provinces, 160 (6.36%) to municipalities of more than 20,000 inhabitants, and, on the other hand, 2334 (92.84%) corresponded to municipalities of 20,000 or less inhabitants. That is, at least in the mid-20th century, when malaria was clearly diminishing in all of Spain, the disease was concentrated at above 90% of the cases in rural areas, away from the big medical centers and close to agricultural areas where the presence of unhealthy liquid collections was frequent.

If we compare the surface occupied in each region by these unhealthy liquid collections with the percentage of malarial municipalities, no statistically significant relation is obtained (R-sq = 0.26). However, if we delete the data of Extremadura from the scatterplot (Figure 2b), a coefficient of determination R-sq = 0.75 is obtained. Apart from Extremadura, Western Andalusia outstands for having the largest surface of malarial wetlands, and Levante and La Mancha for having a greater degree of spreading of the disease in relation to the surface occupied by unhealthy liquid collections. In order to determine whether it is possible to establish a linkage between the presence of unhealthy liquid collections and the degree of endemicity of the disease, we correlated these variables. The number of diseased people and deaths and the surface occupied by unhealthy liquid collections in 1913 and 1916 show an R-sq of 0.34 and 0.27, respectively. Levante and Extremadura showed outlier behavior with respect to the rest of the Spanish regions. Levante and Extremadura were excluded from the analysis and, as a result, the coefficient of determination R-sq increased up to 0.87 (Figure 3c). Concurrently, if only Extremadura is removed in relation to the number of deaths, then an R-sq of 0.78 is obtained (Figure 3d).

The differentiated behavior of Extremadura could be linked to worse hygiene-health conditions than those of the rest of Spain, especially in the north of Badajoz [32]. In the case of Levante, there is a greater number of diseased people in relation to the surface of wetlands, which could be related to the existence of vectors different from those of the rest of the country. Currently, the only vector still widely spread in Spain capable of transmitting the disease is *Anopheles atroparvus* [1]. However, there is another vector related to malaria in Spain (*Anopheles labranchiae*) which disappeared from the Spanish Southwest in 1973 [1,33] due to the abandonment of ricefields [34], the use of pesticides and the improvement of water drainage [33]. *A. labranchiae* is a markedly anthropophilic species, which was especially abundant in 1946 in the provinces of Murcia and Alicante [35].

**Figure 5:**
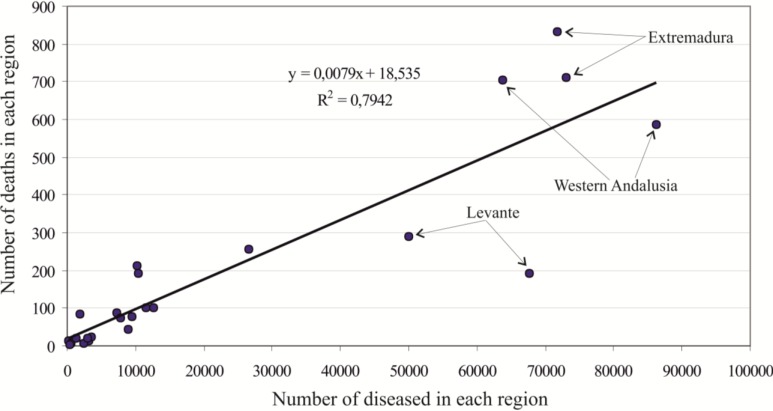
Scatterplot of the number of deaths (1913 and 1916) in each Spanish region.

In order to determine how distant the behavior of Levante and Extremadura are from the general behavior of the rest of the Spanish regions, we jointly presented the number of diseased people and deaths at the regional level in 1913 and 1916 in a scatterplot (Figure 5).

The coefficient of determination R-sq is 0.79 (although it goes up to 0.90 only for the year 1913). Despite including most of the unhealthy water bodies of Spain, the behavior of Western Andalusia is similar to that of the rest of the regions.

**Figure 6:**
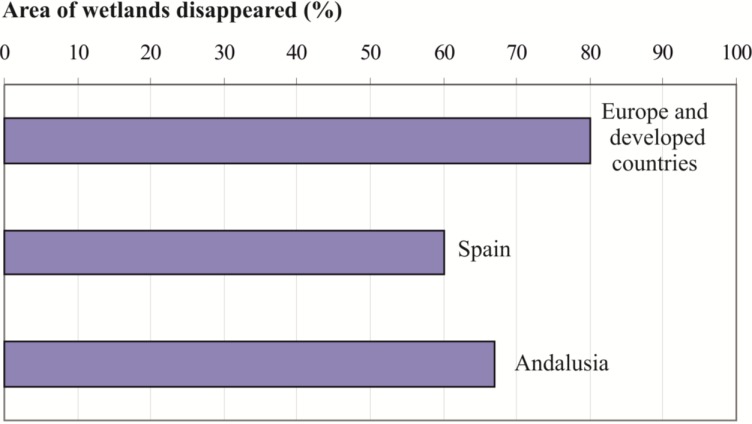
Estimates of the surface of wetlands cleared, according to different authors.

Since throughout the 20th century there has been a progressive decrease in the number of deaths by malaria in Spain, we want to investigate what happened during this time to the unhealthy liquid collections. For this, we represent (Figure 6), in a comparative way, the estimations of the percentage of wetlands throughout the 20th century in Europe and developed countries [36], in Spain [37] and Andalusia [38].

Although there are not many data [38], in Europe and other developed regions it is estimated that the surface of wetland irreversibly removed is 80% [36]. In Spain, a loss of 60%–70% is estimated, mostly during the second half of the 20th century [37]. In Andalusia, around 51% of the wetlands were lost between the 19th century and the 1970s, which would represent an approximate area of 130,000 ha, distributed about 120 missing wetlands [38]. That is, it is estimated that in Andalusia half of the wetlands present in the late 19th century have disappeared, which means that their surface would have been reduced to 33% of their original surface.

In the particular case of the province of Huelva, one of the last Spanish cores where cases of malaria appeared until 1961, peaty lagoons decreased 88.2% [29]. This case is especially interesting since it is one of the most endemic areas of malaria of all Spain, where the process of reduction of these wetlands was framed within a forestry project aimed to desiccate the lagoons in order to restrain the reproduction of *Anopheles atroparvus*, as stated in some documents of that time (Table A1) and more recent studies [14,29,39]. After analyzing the causes of the desiccation of the lagoons, a synergic effect was observed between the measures developed by humans to perform such desiccation [40,41] and the aspects linked to the end of the Little Ice Age in South Spain and to the Climate Change of the 19th and 20th centuries [29,39]. Therefore, we will analyze the changes in the trends of the main climate variables.

**Figure 7:**
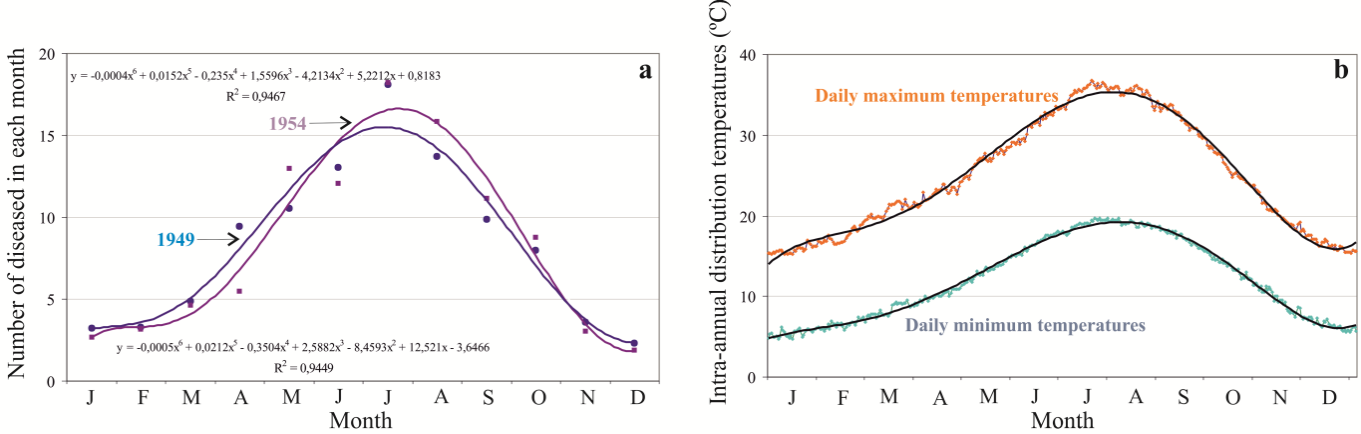
(**a**) Monthly percentage of the distribution of diseased people in 1949 and 1954 and their polynomial functions. (**b**) Inter-annual distribution of the minimum and maximum average temperatures between 1951 and 2001 in Southwestern Spain (Seville).

The geographical and seasonal distribution of many infectious diseases is linked to climate [27]. After studying the monthly distribution of the number of diseased people we found a clearly seasonal pattern (Figure 7); this is regardless of the fact that there are years with many deaths, like 1949 (33,919 deaths), or few, like 1954 (3971 deaths). Figure 7a shows an almost symmetric bell with values below 5% in the winter months, increasing in spring, with a maximum in July (18%), and decreasing in autumn. These values may be compared with the seasonal behavior of the minimum and maximum average temperatures (Figure 7b).

**Figure 8:**
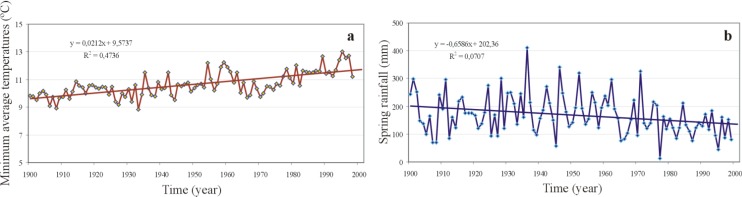
(**a**) Inter-annual variations of the minimum average temperatures in Seville in the 20th century. (**b**) Inter-annual variation of spring rainfall in the southwest of Spain in the 20th century.

The comparison of the patterns of the seasonal distribution of the percentage of diseased people and intra-annual temperatures is clearly parallel. If these results are compared with the density of the vector in the southwest of Spain (where the data shown in Figure 7b are from), *Anopheles atroparvus* is distributed from the beginning of June to the end of September in the province of Huelva [42]. This is completely in line with Figure 7, since the distribution of temperatures is essential for the development of both the vector and the parasite [42]. There is evidence that climate change provokes situations that affect the development of the vector and the current transmission and geographical distribution of the disease, in relation to both thermal variables and rainfall [43]. Therefore, we are going to investigate the inter-annual variations of the minimum average temperatures and the variations in spring rainfall in the southwest of Spain, where most of the unhealthy wetland surface is historically concentrated (Figure 8).

In the Iberian Peninsula, the analysis of temperatures shows a warming in the 20th century with two periods: one in the first half of the century and a second one from the 1970s [44]. This thermal increase is especially sharp in the minimum average temperatures (Figure 8a). Rainfall can also be a limiting factor for the populations of mosquitoes and there is some proof of the reduction in transmission associated to decadal rainfall decreases [45]. Although total annual rainfall does not show statistically significant trends throughout the 20th century, the analysis of seasonal rainfall reveals a marked trend to the decrease of spring rainfall during the 20th century in the southwest of Spain. Specifically, the decrease of spring rainfall throughout the 20th century is one third of the total spring rainfall (60 L/m^2^). Although this decrease is compensated by an increase in the rainfall of other seasons, it involves a real loss of supply for the wetlands in the period during which the vector develops and grows.

In order to grow inside the mosquito, *Plasmodium falciparum* and *P. vivax* require a minimum temperature of 19 ºC and 16 ºC, respectively [1,44]. Therefore, the thermal climate trends from the early 20th century could have favored the development of the disease, in the same way as it is expected to happen according to the forecast for the 21st century. In contrast, the sharp desiccation of many wetlands along with a decrease of the spring rainfall played a relevant role in the regression of autochthonous malaria in Spain. In the particular case of the province of Huelva (Southwest Spain), a program was designed to desiccate the wetlands where the mosquito developed and grew (see Table A1). The desiccation of these wetlands played a relevant role in the elimination of one of the last endemic foci of malaria in Spain [14,29]. Despite the known causal links between climate and transmission dynamics of malaria, there is great uncertainty about the potential impact of climate change on this disease. This is due to the lack of detailed, concurrent, historical observations on climate and malaria, and to the complexity of the dynamics of malaria influenced by socioeconomic factors, immunity, drug resistance, *etc*. [45].

## 4. Conclusions

The use of the GIS-supported IDW method to visualize the spatial occurrence in the analysis of the historical data of malaria in Spain allows us to conclude that the distribution was not homogenous. The main endemic foci, serious and recurrent, were Extremadura (especially the north of the region), the west side of Andalusia, La Mancha and Levante (including the current province of Murcia). The existence of these foci in the early 20th century is mostly linked to rural and socioeconomically depressed areas, to the presence of *Anopheles labranchiae* in the Spanish Mediterranean coast and to the existence of large areas occupied by wetland and other unhealthy water bodies. These unhealthy water bodies, which act as reservoirs of the disease, are situated mostly in Western Andalusia (208,527.0 ha) and to a lesser extent in La Mancha (58,131.5 ha) and Levante (33,445.0). The data of diseased people and deaths in the early 20th century show the important role they played in the generation of seriously endemic foci of malaria, besides the role played also by the hygiene-health conditions and the existence of different vectors. It must be taken into account that in Andalusia these malarial foci were favored by periods of great rainfall during the Little Ice Age, including the third and last one, which occurred in the late 19th century [46].

Already in the 20th century, and especially from the 1950s, in the specific case of Western Andalusia, these unhealthy flooded areas were slowly desiccated. In the United Kingdom, the diminution of malaria, in the late 19th century, is also linked to the reduction of wetlands, among other factors [47].

The existence of important population flows, along with the proximity of the African coast, involve additional factors to be considered when analyzing the risk of re-emergence of malaria in Spain. In the early 20th century, the movements of migrants from the Spanish Southeast to Argelia [31] favored the spread of the disease. Later, these population flows were one of the factors that enhanced the epidemic of the Spanish Civil War [13].

The past distribution of autochtonous malaria in Spain was influenced by a variety of different driving forces. On the one side we can find different vectors and on the other side we have the influence of socio-economic conditions and the existence of water bodies which supports the reproduction of the transmission vector of the disease. The important role of the presence of unhealthy water bodies in the past will allow us to better understand the processes which could lead to a re-emergence of malaria in countries where the disease disappeared in the 20^th^ century as well as in those countries where it is currently present.

In our opinion, all the factors mentioned lead to think of a very low risk of re-emergence in Spain [1], limited only to small outbreaks of introduced malaria, of which one case has already been reported in Spain [6], as long as the socioeconomic and hygiene-health conditions of the country do not deteriorate. However, the history of the disease proofs the importance of maintaining vigilance [3,14] and increasing research, especially on marshland areas [48].

## Appendix

**Table A1:** Text from the 19th and mid 20th centuries elaborated by the people in charge of the desiccation of coastal lagoons from the province of Huelva.

Year	Description	Author
1848	*“…in high places, brain, chest and throat disorders are very common; intermittent, tertian and quartan fevers are present in this places specially in summer, and intermittent fevers in flat terrain” (sic)*	Madoz, P. *Diccionario Geográfico-estadístico-histórico de España y sus posesiones de Ultramar*. Biblioteca Santa Ana: Almendralejo, Spain, 1848.
1890	*"... additionally, a good number of infected lagoons and puddles are scattered on it, which fill up the environment with unhealthy malarial vapours"*	Heraso, J. Estudio sobre la fijación de las dunas situadas en el término municipal de Almonte, provincia de Huelva. Primera Parte. *Revista de Montes* 1890, *322*, 281–287.
1936	*"... that, once reafforested, this area —which is currently arid and unhealthy due to malaria— can be turned into a magnificent and healthy pine forest..." “... "... thus providing the region with new benefits"*	Kith, M. Propuesta de ampliación del proyecto de fijación y repoblación de las Dunas de Almonte. V División Hidrológico-Forestal del Guadalquivir, 1936, 15 p.
1941	*“Being the property located in a malaria infected region, we consider necessary to carry out works for its sanitation and taking all the preventive measures in order to reducing as far as possible the risk of infection of the employees engaged in the works”*	De La Lama, G. Memoria de reconocimiento y propuesta de trabajos de la finca “Coto Ibarra” (Informe Técnico). Patrimonio Forestal del Estado, 1941, 45 p.
1941	*"... most of them keep holding water during the summer, but they greatly facilitate the reproduction of mosquitoes (anopheles) and the resulting spread of malaria".*	De La Lama, G. Memoria de reconocimiento y propuesta de trabajos de la finca “Coto Ibarra” (Informe Técnico). Patrimonio Forestal del Estado, 1941, 45 p.
1951	*"... in that huge and depressing loneliness that was only disturbed by the buzzing of the thick cloud of mosquitoes, potential carriers of malaria, that enveloped us both horses and riders"*	De La Lama, G. Diez años de trabajos forestales. *Revista de Montes* 1951, *39*, 195–201.
